# Exploration of anti-cancer effects and mechanisms of Zuo-Jin-Wan and its alkaloid components in vitro and in orthotopic HepG2 xenograft immunocompetent mice

**DOI:** 10.1186/s12906-017-1586-6

**Published:** 2017-02-20

**Authors:** Shun-Ting Chou, Chien-Yun Hsiang, Hsin-Yi Lo, Hui-Fen Huang, Ming-Tsung Lai, Ching-Liang Hsieh, Su-Yin Chiang, Tin-Yun Ho

**Affiliations:** 1 Graduate Institute of Chinese Medicine, China Medical University, 91 Hsueh-Shih Road, Taichung, 40402 Taiwan; 2 Department of Microbiology, China Medical University, Taichung, 40402 Taiwan; 3 School of Post-baccalaureate Chinese Medicine, Tzu Chi University, Hualien, 97004 Taiwan; 4 School of Medicine, Chung-Shan Medical University, Taichung, 40201 Taiwan; 5 Department of Pathology, Chung-Shan Medical University Hospital, Taichung, 40201 Taiwan; 6 Graduate Institute of Integrated Medicine, China Medical University, Taichung, 40402 Taiwan; 7 Department of Chinese Medicine, China Medical University Hospital, Taichung, 40447 Taiwan; 8 Department of Health and Nutrition Biotechnology, Asia University, Taichung, 41354 Taiwan

**Keywords:** Zuo-Jin-Wan, Anti-cancer, *Coptis chinensis*, *Evodia rutaecarpa*, Xenograft mouse model

## Abstract

**Background:**

Zuo-Jin-Wan (ZJW), a two-herb formula consisting of *Coptis chinensis* (CC) and *Evodia rutaecarpa* (ER), is commonly used in traditional Chinese medicine for the treatment of cancers. However, the efficacies and mechanisms of ZJW and its alkaloid components on cancers are still unclear.

**Methods:**

Here we investigated the anti-cancer effects and mechanisms of ZJW, CC, ER, berberine, and evodiamine in cells and in intrahepatic xenograft mice.

**Results:**

Treatment of HepG2 cells with ZJW, CC, ER, berberine, and evodiamine significantly displayed cytotoxic effects in a dose- and time-dependent manner. Hierarchical cluster analysis of gene expression profiles showed that CC and ZJW shared a similar mechanism for the cytotoxic effects, suggesting that CC was the active ingredient of ZJW for anti-cancer activity. Network analysis further showed that c-myc was the likely key molecule involved in the regulation of ZJW-affected gene expression. A human hepatoma xenograft model was established by intrahepatic injection of HepG2 cells containing nuclear factor-κB-driven luciferase genes in immunocompetent mice. In vivo bioluminescence imaging showed that cells had been successfully transplanted in mouse liver. Oral administration of ZJW for 28 consecutive days led to a significant decrease in the accumulation of ascites, the ratio of tumor-to-liver, and the number of transplanted cells in livers.

**Conclusions:**

In conclusion, our findings suggested for the first time that ZJW significantly suppressed human cancer cell growth in orthotopic HepG2 xenograft-bearing immunocompetent mice. Moreover, c-myc might play a potent role in the cytotoxic mechanisms of ZJW, CC, ER, berberine, and evodiamine.

## Background

Hepatocellular carcinoma (HCC) is the fifth most common cancer and the third leading cause of cancer death worldwide. Over 80% of newly diagnosed HCC cases occur in developing countries. In China, more than 350,000 patients are diagnosed with HCC each year, representing about one-half of all new HCC cases worldwide. In Taiwan, HCC is the second leading cause of cancer death [1]. Most patients with HCC are not suitable for surgical resection, and fewer than 20% patients respond to conventional chemotherapy. Thus, it is imperative to develop more effective therapies for HCC treatment [2, 3].

Many herbal formulations in traditional Chinese medicine (TCM) are used alone or as an adjuvant therapy to conventional radiation therapy, chemotherapy, or surgical resection for the treatment of cancer [4–6].

Zuo-Jin-Wan (ZJW) is a well-known two-herb Chinese medicinal formula that consists of *Coptidis rhizoma* and *Evodiae fructus* at a ratio of 6:1 (w/w) [7]. *Coptidis rhizoma*, also known as Huang-Lian, is the dried rhizome of *Coptis chinensis* Franch (CC). CC is used in TCM to treat diabetes, inflammation, vomiting, diarrhea, as well as respiratory disorders [8–10]. *Evodiae fructus*, also known as Wu-Chu-Yu, is the immature fruit with stems of *Evodia rutaecarpa* Benth (ER). ER is widely used in TCM for the treatment of inflammation, headache, diarrhea, and hypertension [4, 11, 12]. Our previous in vitro study revealed that ZJW inhibits the activities of activator protein 1 (AP-1) and nuclear factor-κB (NF-κB), as well as cellular transformation in HepG2 cells [13]. Lately, ZJW has been shown to induce apoptosis in HepG2 cells through a mitochondrial-mediated and caspase-dependent pathway [14]. In addition, an in vivo study found that ZJW not only inhibits the growth of transplanted murine sarcoma S180 tumor cells in Chinese Kunming mice, but also prolongs the life span of S180-bearing mice [15]. ZJW was reported to reverse multidrug resistance of human colorectal cancer by increasing the sensitivity of multidrug-resistant cells to chemotherapeutic drugs and decreasing the P-glycoprotein level in vitro and in vivo [16, 17]. ZJW also suppresses the growth of both human colorectal cancer xenograft in nude mice and 4 T1 mouse breast cancer in the BALB/c mice [16, 18]. However, the anti-cancer effects of ZJW against human hepatoma cells in either nude mice or immunocompetent mice remain to be elucidated.

So far, the anti-cancer effects of botanicals and their components are mainly studied in subcutaneous tumor transplantation models in immunodeficient mice. Only a very few intrahepatic xenograft models of HCC are available [19]. The main advantages of using subcutaneous models include easy access for tumor mass measurement and easy monitoring of tumor development and treatment efficacy. However, these subcutaneous tumors usually grow as encapsulated, poorly vascularized masses [20]. Several studies have suggested that neighboring cells and extracellular matrix components profoundly affect the tumor cell phenotype, and further emphasized the need to examine the antitumor activities of agents in the context of appropriate tumor environment [19]. This prompts us to develop an orthotopic intrahepatic HepG2 xenograft mouse model to study the anti-cancer effects of ZJW.

The anti-cancer effects of ZJW in an orthotopic intrahepatic HepG2 xenograft model and the molecular mechanisms underlying the anti-cancer activities of ZJW and its alkaloid components were investigated in this study. HepG2/ NF-κB/Luc cells were injected into the left lateral hepatic lobe of mice to establish the intrahepatic tumor mode. ZJW was orally given to mice for 28 consecutive days. The anti-cancer effects and mechanisms were then analyzed by immunohistochemical examination and transcriptomic analysis.

## Methods

### Chemicals and cell line

All chemicals were purchased from Sigma (St. Louis, MO, USA) unless indicated. Evodiamine (E3531) and berberine chloride (B3251) were dissolved in dimethyl sulfoxide at 100 mM and 33 mM, respectively. Recombinant HepG2/NF-κB/Luc cells containing the luciferase genes driven by NF-κB-responsive elements were constructed as described previously [21]. D-Luciferin was purchased from Xenogen (Hopkinton, MA, USA) and dissolved in phosphate-buffered saline (PBS). Mouse monoclonal antibody against luciferase was purchased from Santa Cruz (Santa Cruz, CA, USA).

### Preparation and quality analysis of aqueous extracts of ZJW, CC, and ER

ZJW is a two-herb Chinese medicinal formula which consists of CC and ER at a ratio of 6:1 by weight. The concentrated herbal powders of ZJW, CC, and ER were purchased from Sun Ten Pharmaceutical Co. (Taipei, Taiwan), a renowned GMP manufacturer of concentrated herbal extracts by both Taiwanese and Australian authorities. Chinese medicinal herbs and formulae are usually prepared by boiling the herbs in water in TCM. Distilled water was therefore used for the extraction purpose in this study. To prepare the aqueous extracts of ZJW, CC, and ER for in vitro studies, individual concentrated herbal powder (15 g) was soaked in 75 mL double-distilled water, boiled for 30 min, and then centrifuged at 1,000 rpm for 20 min. The resulting supernatant was collected and store at −30 °C. The recovery of ZJW, CC, and ER was approximately 15% by weight. The contents of standard compounds (berberine and evodiamine) of CC and ER were further evaluated by high-performance liquid chromatography (HPLC).

HPLC was performed on a Waters 2695 HPLC instrument and a 2996 Photo Diode Array Detector (Waters, Milford, MA, USA). The column used here was Atlantis column (C-18, 250 mm × 4.6 mm) (Waters, Milford, MA, USA). Mobile phase for CC and berberine was performed using acetonitrile, potassium dihydrogen phosphate, and sodium lauryl sulfate. The flow rate was 1.0 mL per min and the detection was under the absorption wavelength of 345 nm within 20 min. Mobile phase of ER and evodiamine was performed using Solvent A (water, acetic acid, oxolane) and B (acetonitrile) which were mixed equally. The flow rate was 1.0 mL per min and the detection was under the absorption wavelength of 225 nm within 20 min.

### Cell toxicity assay

HepG2/NF-κB/Luc cells were maintained in Dulbecco’s modified Eagle’s medium (DMEM) (Life Technologies, Gaithersburg, MD, USA) supplemented with 10% fetal bovine serum (Hyclone, Logan, Utah, USA). Cells in logarithmic growth phase were treated with different concentrations of ZJW, CC, ER, berberine, and evodiamine for 24, 48, or 72 h. Cell viability was then determined by a 3-[4,5-dimethylthi- azol-2-yl]-2, 5-diphenyltetrazolium bromide (MTT) colorimetric assay. Cell viability (%) was calculated as [(absorbance of drug-treated cells-absorbance of medium only)/(absorbance of solvent-treated cells-absorbance of medium only)] × 100%. Toxic concentration at 50% (TC_50_) was determined as the concentration of drug required to reduce cell viability by 50% relative to solvent-treated cells.

### Microarray analysis

Total RNA was extracted from HepG2/NF-κB/Luc cells following treatment with ZJW, CC, ER, berberine, or evodiamine at TC_50_ for 48 h by RNeasy Mini kit (Qiagen, Valencia, CA, USA). Microarray analysis was performed as previously described [22, 23]. Briefly, fluorescence-labeled RNA targets were prepared from total RNA using a MessageAmp™ RNA kit (Ambion, Austin, TX, USA) and Cy5 dye (Amersham Pharmacia, Piscataway, NJ, USA). Triplicate fluorescent targets were hybridized to the Human Whole Genome OneArray™ (Phalanx Biotech Group, Hsinchu, Taiwan) and scanned using an Axon 4000 scanner (Molecular Devices, Sunnyvale, CA, USA). Cy5 fluorescent intensity of each spot was analyzed using Genepix 4.1 software (Molecular Devices, Sunnyvale, CA, USA). Signal intensity of each spot was normalized by R program in limma package using quantile normalization. Hierarchical clustering analysis of down-regulated genes was performed and displayed using the TIGR Multiexperiment Viewer (http://mev.tm4.org/). An interaction network for genes with fold changes ≤ −2.0 was generated using Transcription Regulation algorithm in MetaCore™ Analytic suit (GeneGo Inc., St. Joseph, MI, USA).

### Quantitative real-time polymerase chain reaction (qPCR)

The expression level of eukaryotic translation initiation factor 4A isoform 2 (EIF4A2) was validated by qPCR. Total RNA was reverse-transcribed at 37 °C for 120 min using High Capacity cDNA Reverse Transcription kit (Applied Biosystems, Foster City, CA, USA). qPCR was performed by the following condition: 10 min at 95 °C; 40 cycles of 15 s at 95 °C and 1 min at 60 °C. Each assay was run on an Applied Biosystems 7300 Real-Time PCR system in triplicates. Fold changes were calculated using the comparative CT method [24]. Glyceraldehyde-3-phosphate dehydrogenase (GAPDH) gene was used as an endogenous control. The primer set for each gene is as follows. EIF4A2 forward primer 5′-GGAGGAGATGCCCATGAATG-3′; EIF4A2 reverse primer 5′-GTGATCGCCTATTCAGCAACAG-3′; GAPDH forward primer 5′-TCACCCACACTGTGCCCATCTATGA-3′; GAPDH reverse primer 5′-GAGGAAGAGGATGCGGCAGTGG-3′.

### Intrahepatic tumor models

Mouse experiments were conducted with the ethics approval from China Medical University Animal Care and Use Committee (Permission No. 2016–034). Female ICR mice (4–6 weeks old, 30 g) were purchased from National Laboratory Animal Center (Taipei, Taiwan). Mice were anesthetized intraperitoneally with 80 mg/kg ketamine and 10 mg/kg xylazine. Anesthesia was maintained using a snout cone. A left subcostal incision of abdomen was made. HepG2/NF-κB/Luc cells (1 × 10^6^ cells in 50 μl DMEM) were injected into the left lateral hepatic lobe. Hemostasis was maintained by applying direct pressure over the injection site using a 26-gauge needle. Peritoneum and skin were then closed with 6.0 absorbable Vicryl sutures. Mice were allowed to recover and then returned to the animal housing chambers.

### Animal experiment

Three days after tumor cell implantation, in vivo bioluminescence imaging was performed as described previously [25]. Briefly, mice were anesthetized by isoflurane, intraperitoneally given with 150 mg/kg D-luciferin, and imaged for 1 min with an IVIS Imaging System^®^ (Xenogen, Hopkinton, MA, USA). Photons emitted from the bodies were quantified as total photons/s by the Living Image^®^ software (Xenogen, Hopkinton, MA, USA). A total of 36 mice was equally divided into treatment groups (ZJW, 200 mg concentrated powder/kg) and control groups (PBS), according to imaging data. At intervals of 7-, 14-, or 28-day after daily treatment with ZJW or PBS, mice were sacrificed to examine the therapeutic effects of ZJW. The degree of ascites was scored as follows: 1, no ascites; 2, minimal ascites; 3, moderate ascites; 4, severe ascites. All animals were allowed free access to food and water during the experiment. In addition, all mice were kept under observation and weighed during the experiment.

### Histopathological examination

Mice were sacrificed by CO_2_ asphyxiation two days after the last administration. Livers were dissected and the weight of liver was measured. Liver specimens were then fixed in buffered formalin and stained with hematoxylin and eosin for histological examination. Histopathological evaluation was performed blinded by an experienced pathologist from the Department of Pathology at Chung Shan Medical University Hospital (Taichung, Taiwan).

### Immunohistochemical staining

Sections of 5 μm were deparaffinized in xylene and rehydrated in graded alcohol. Antigen retrieval was performed with sodium citrate buffer (10 mM sodium citrate, 0.05% Tween 20, pH 6.0) at 60 °C overnight. The nonspecific binding was blocked with 1% (w/v) bovine serum albumin at room temperature for 1 h. Sections were incubated with mouse monoclonal antibody against luciferase at 1:50 dilution at 4 °C overnight. Sections were then incubated with secondary antibody at room temperature for 1 h and stained with 3,3′-diaminobenzidine (HistoStain^®^ Plus kit, Invitrogen, Carlsbad, CA, USA). Finally, the immunostained cells were visualized under a light microscope.

### Statistical analysis

Data are expressed as mean ± standard deviation and tested for normality by Shapiro-Wilk and Kolmogorov-Smirnov tests, using the Statistical Analysis Software (SAS) package (Version 9.1, SAS Institute, Cary, NC, USA). Normal distribution data were analyzed by one-way ANOVA followed by the Scheffe’s test. Non-normal distribution data were analyzed by nonparametric Wilcoxon rank-sum test, as provided in PROC NPAR1WAY. Statistical significance was set at *p* < 0.05.

## Results

### Determination of alkaloid ingredients in CC and ER

**Fig. 1:**
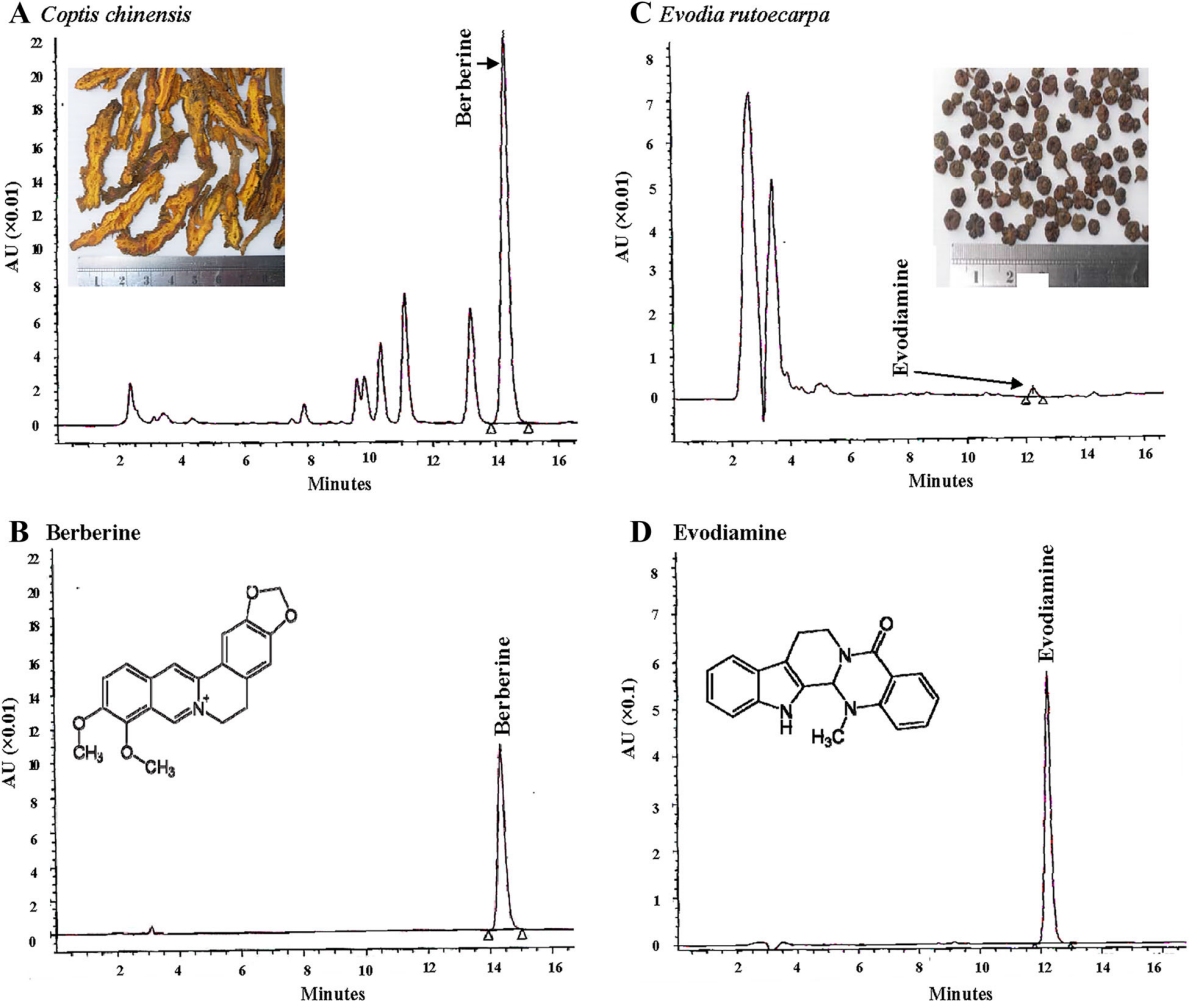
HPLC chromatographs of CC (**a**), berberine (**b**), ER (**c**), and evodiamine (**d**). Photographs and chemical structures of CC, ER, berberine, and evodiamine are shown in each chromatograph

The contents of berberine and evodiamine in CC and ER, respectively, were analyzed by comparing the retentive time with standard samples. HPLC analysis showed that the retention time of berberine and evodiamine was 14.3 min and 12.2 min, respectively (Fig. 1). The contents of berberine and evodiamine in CC and ER were 5.5 and 0.0085%, respectively.

### Cytotoxicities of ZJW, CC, ER, berberine, and evodiamine in HepG2/NF-κB/Luc cells

**Fig. 2:**
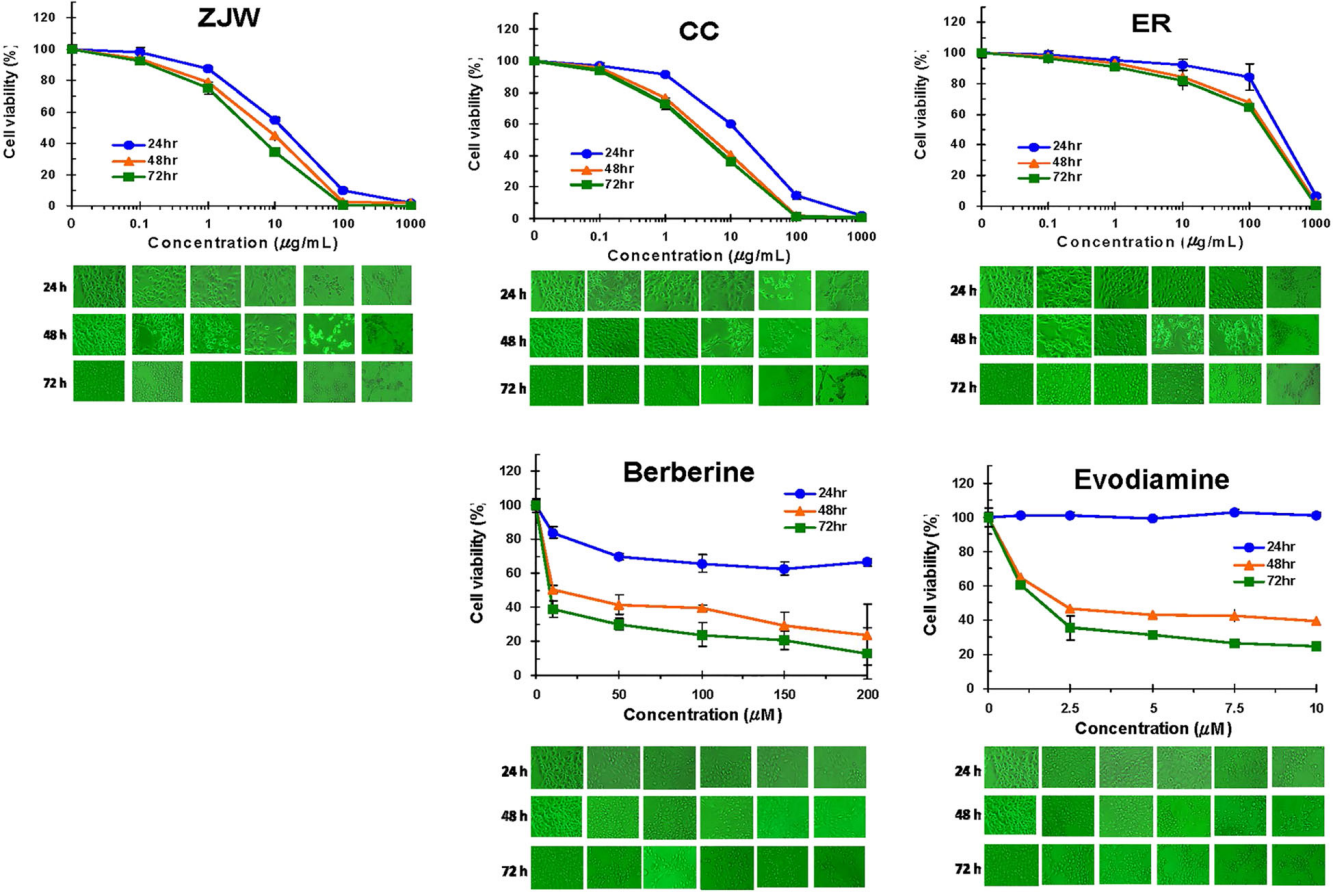
Cytotoxicities of ZJW, CC, ER, berberine, and evodiamine in HepG2/NF-κB/Luc cells. HepG2/NF-κB/Luc cells were treated with various concentrations of drugs for 24, 48, or 72 h. Cell viability was evaluated by MTT assay. Values are mean ± standard deviation of three independent experiments. Cellular morphology is shown at the bottom

Cell viability of HepG2/NF-κB/Luc cells exposed to various amounts of ZJW, CC, ER, berberine, or evodiamine for 24, 48, or 72 h was tested by MTT assay. ZJW, CC, ER, and berberine exhibited cytotoxicities in HepG2/NF-κB/Luc cells in a dose- and time-dependent manner (Fig. 2). Although evodiamine displayed no cytotoxic effect at the highest dose (10 μM) after 24 h treatment, it exhibited cytotoxicity at 48 h and 72 h. The TC_50_ values at 48 h were 7.7 μg/mL for ZJW, 4.3 μg/mL for CC, 320 μg/mL for ER, 5.9 μg/mL (15.3 μM) for berberine, and 0.7 μg/mL (2.3 μM) for evodiamine.

### Microarray analysis of HepG2/NF-κB/Luc cells treated with ZJW, CC, ER, berberine, and evodiamine

**Fig. 3:**
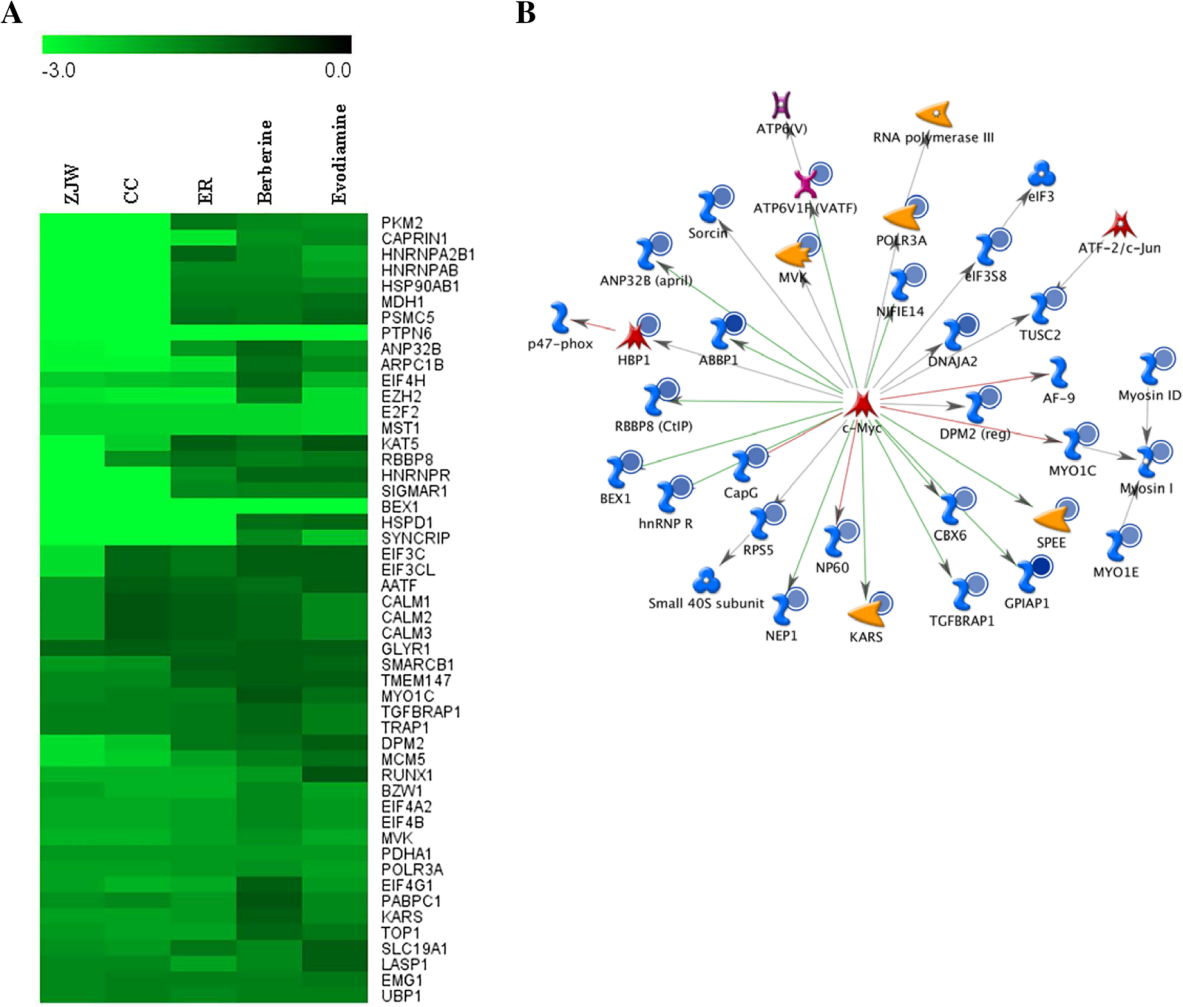
Microarray analysis of HepG2/NF-κB/Luc cells treated with ZJW, CC, ER, berberine, and evodiamine at TC_50_ for 48 h. **a** Hierarchical clustering analysis of gene expression profiles altered by ZJW, CC, ER, berberine, and evodiamine. Normalized log_2_ expression values are color-coded according to the legend at the top. **b** Network analysis of genes regulated by ZJW, CC, ER, berberine, and evodiamine in HepG2/NF-κB/Luc cells. Gene commonly down-regulated by 2-fold by ZJW, CC, ER, berberine, and evodiamine were selected for the generation of network

To explore the cytotoxic mechanisms of ZJW, CC, ER, berberine, and evodiamine, microarray analysis was conducted in HepG2/NF-κB/Luc cells exposed to compounds at TC_50_ for 48 h. In a total of 30,967 transcripts, the expressions of 327, 237, and 116 genes were down-regulated (fold changes ≤ −2.0) by ZJW, CC, and ER, respectively, while the expressions of 228, 171, and 57 genes were up-regulated (fold changes ≥ 2.0) by ZJW, CC, and ER, respectively. There were 82 and 78 genes altered by berberine and evodiamine, respectively, with fold changes ≤ −2.0. The expressions of 71 and 62 genes were up-regulated by berberine and evodiamine, respectively. There were three genes commonly upregulated by ZJW, CC, ER, berberine, and evodiamine. These genes included granulocyte colony stimulating factor 3, long intergenic non-protein coding RNA 265, and sine oculis homeobox homolog 2. However, a total of 64 genes were commonly downregulated by ZJW, CC, ER, berberine, and evodiamine. Therefore, we selected genes commonly down-regulated by ZJW, CC, ER, berberine, and evodiamine for hierarchical clustering analysis. As shown in Fig. 3a, CC shared a similar gene expression profile with ZJW, suggesting that ZJW and CC shared similar mechanisms for cytotoxic effects. The expression level of EIF4A2 was further validated by qPCR. Table 1 shows that ZJW, CC, ER, berberine, and evodiamine downregulated the expression of EIF4A2 gene, which was consistent with microarray data. Genes with fold changes ≤ −2.0 after drug treatment were further selected to construct the interaction network using Pathway Edition software. Among 64 genes, 35 genes were directly connected to c-myc, suggesting that the expression of these genes was regulated by c-myc (Fig. 3b). Moreover, c-myc seemed to be in the central part of the network, suggesting that c-myc was the likely key molecule involved in the regulation of ZJW-, CC-, ER-, berberine-, and evodiamine-affected gene expression.

**Table 1:** Verification of gene expression by qPCR

Gene	Sample	Average C_T_	Average C_T_ of GAPDH	ΔC_T_^a^	ΔΔC_T_^b^	Fold change
EIF4A2	Mock	20.08 ± 0.26	32.36 ± 0.25	−12.28 ± 0.26	0 ± 0.26	1
	ZJW	22.97 ± 0.13	35.17 ± 0.1	−12.2 ± 0.13	0.08 ± 0.13	−1.05
	CC	22.78 ± 0.11	34.39 ± 0.09	−11.61 ± 0.11	0.67 ± 0.11	−1.59
	ER	20.79 ± 0.15	30.88 ± 0.05	−10.09 ± 0.15	2.19 ± 0.15	−4.55
	Berberine	19.32 ± 0.11	30.25 ± 0.1	−10.93 ± 0.11	1.35 ± 0.11	−2.56
	Evodiamine	19.84 ± 0.02	30.94 ± 0.02	−11.1 ± 0.02	1.18 ± 0.02	−2.27

^a^The ΔC_T_ value is determined by subtracting the average C_T_ value of GAPDH gene from the average C_T_ value of target gene. The standard deviation of the difference is calculated from the standard deviations of the target gene and GAPDH gene

^b^The ΔΔC_T_ value is determined by subtracting the ΔC_T_ value of mock group from the ΔC_T_ value of treatment group. This is a subtraction of an arbitrary constant, so the standard deviation of ΔΔCT is the same as the standard deviation of ΔC_T_ value

^c^Fold change is presented as 2^-ΔΔCT^ if the value is ≥ 1, or as −1/2^-ΔΔCT^ if the value is <1

### Anti-cancer effects of ZJW in an orthotopic intrahepatic xenograft model

**Fig. 4:**
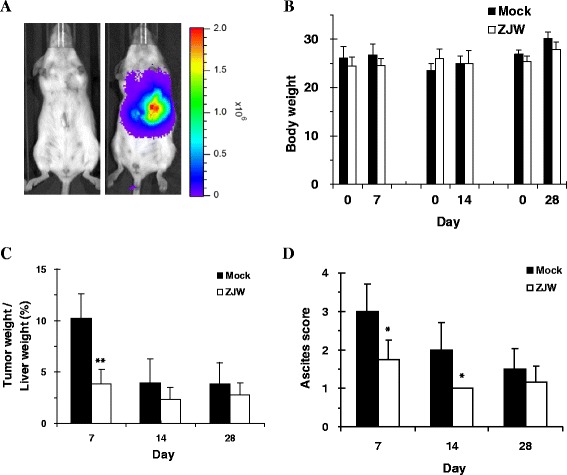
Effect of ZJW in an orthotopic intrahepatic xenograft mouse model. Mice were injected with HepG2/NF-κB/Luc cells. Three days after tumor cell implantation, mice were subjected to image for bioluminescent signals (**a**) and divided into ZJW treatment groups and mock groups. Left picture represents no cell transplantation. Right picture represents cell transplantation. The color overlay on the image represents photons/s emitted from the body, as indicated by the color scales. Photos are representative images (*n* = 18). Mice were then received ZJW (200 mg/kg) or PBS by gavages every day. At 7-, 14-, or 28-day intervals, mice were sacrificed to measure the body weight (**b**), tumor-liver ratio (**c**), and ascites scores (**d**). Values are mean ± standard deviation (*n* = 6). *p < 0.05, **p < 0.01, compared with mock

HCC xenograft model was established by direct intrahepatic injection of HepG2/NF-κB/Luc cells into mice. Immunocompetent ICR mice were treated with 0.2 mL of pristane 7 d before inoculation with HepG2/NF-κB/Luc cells. Three days after tumor cell implantation, in vivo bioluminescence imaging was performed to verify the transplantation. Imaging data were further used as criteria for selecting and grouping. As shown in Fig. 4a, an intense signal was emitted in the upper abdominal region of mice. All mice injected with HepG2/NF-κB/Luc cells displayed obvious bioluminescent intensity in the upper abdominal region, and mice with luminescent intensity ≥ 1 × 10^7^ photons/s were selected. Thirty-six mice were divided into treatment and control groups. We treated tumor-bearing mice with 200 mg/kg ZJW (concentrated powder) or PBS by gavages. At 7-, 14-, or 28-day intervals after daily treatment with ZJW or PBS, mice were sacrificed to evaluate the therapeutic effects of ZJW. There were no significant differences in body weight among ZJW-treated group and control group at the end of the experiment (Fig. 4b). However, tumor-to-liver ratios were decreased in ZJW treatment group at 7 d after treatment (Fig. 4c). In comparison with control group, ascites scores were also significantly decreased in ZJW-treated group at 7 d and 14 d after treatment (Fig. 4d).

**Fig. 5:**
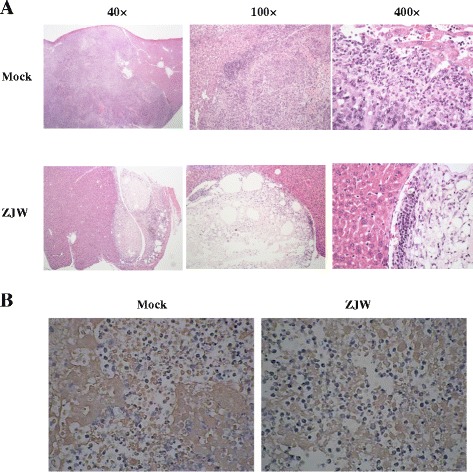
Histopathological examination and immunohistochemical staining of ZJW-treated livers in an orthotopic intrahepatic xenograft mouse model. Mice were injected with HepG2/NF-κB/Luc cells. Three days later, mice were orally given with 200 mg/kg ZJW or PBS for consecutive 7 d. Livers were then collected for histopathological examination (**a**) and immunohistochemical staining with anti-luciferase antibody (**b**). Photos are representative images (*n* = 6). Magnification 400 ×

To confirm the anti-cancer effect of ZJW in livers, liver sections were stained with hematoxylin/eosin or antibody against luciferase. As shown in Fig. 5a, tumor mass and infiltration of inflammatory cells were observed in control group. However, there were several hollows inside the tumor, and the inflammatory cells were located at the margin of tumor mass in ZJW treatment group. In addition, there were brown luciferase-positive cells in the livers in both groups (Fig. 5b). However, the number of luciferase-positive cells was decreased in ZJW treatment group, compared to control group. These findings suggested that oral administration of ZJW exhibited anti-cancer effects by decreasing the weight of tumor mass and the accumulation of ascites in an orthotopic intrahepatic xenograft model.

## Discussion

Chinese medicinal herbs or formulae have been used to treat human cancers for centuries [4–6]. For example, Radix Paeoniae Rubra, the dried root of *Paeonia lactiflora* Pallas and *Paeonia veitchii* Lynch, has an anti-cancer effect on bladder cancer [26]. Chinese licorice (*Glycyrrhiza uralensis* Fisch.), one of the commonly prescribed herbs in TCM, is associated with immune-modulating and anti-tumor potential [27]. *Rumex hastatus* possesses strong cytotoxic potentials in various cancer cell lines [28]. Chloroform extract and saponins from *Polygonum hydropiper* shows anti-angiogenic and anti-tumor activities in fibroblast NIH/3 T3 cell line [29]. Kuan-Sin-Yin decoction, a popular qi-promoting herbal medicine, is constituted with several herbs known to exhibit immunomodulating or anticancer activity [30]. San-Huang-Xie-Xin-Tang exhibits anti-cancer activities via pattern via p53 signaling, p53 activated, and DNA damage signaling pathways in HepG2 cells [31]. Here we found that treatment of HepG2/NF-κB/Luc cells with ZJW significantly displayed cytotoxicities in a dose- and time-dependent manner. Previous study showed that ZJW inhibits phorbol ester-induced NF-κB and AP-1 activities in HepG2 cells. Moreover, ZJW inhibits phorbol ester-induced hepatocellular transformation by suppressing NF-κB and AP-1 activation without causing cytotoxicity [13]. These findings suggested that ZJW was a potential anti-tumor promoting agent in liver.

MTT assay showed that CC and berberine exhibited comparable TC_50_ values (4.3 and 5.9 μg/mL, respectively) in cells. These data suggested that berberine was the active ingredient of CC for cytotoxicity. However, Lin et al. [32] found that the TC_50_ values of CC and berberine are 20.1 μg/mL and 3.1 μg/mL, respectively, in HepG2 cells after a 48-h treatment. Their data suggested that components other than berberine might play roles in the cytotoxic effects of CC. Further study is needed to clarify the proportion of berberine on the cytotoxicity of CC. In contrast, TC_50_ values differed greatly between ER and evodiamine (320 and 0.7 μg/mL, respectively) in HepG2/NF-κB/Luc cells. A low TC_50_ value of evodiamine suggested that it was more active than ER on the cytotoxic activity. Although this was the first report to show that ER displayed cytotoxicity, Xu et al. [33] showed that evodiamine, the major quinolone alkaloid in ER, exhibits the cytotoxic activity in HepG2 cells. In addition, Wang et al. [34] showed that mixtures of berberine and evodiamine display the highest inhibitory effect on SMMC-7721 cells, compared with berberine and evodiamine used individually 48 h. Moreover, the combined use of berberine and evodiamine significantly enhance the apoptosis of SMMC-7721 cells.

Microarray analysis was performed to elucidate the cytotoxic effects of ZJW and its components. Gene network analysis showed that c-myc played a central role in the cytotoxic effects of ZJW and its ingredients. c-Myc protein, encoded by the c-myc proto-oncogene, is a transcription factor involved in cell proliferation. Over-expression of c-Myc caused by genomic amplification of c-myc proto-oncogene is found in up to 70% of viral and alcohol-related HCC. Also, c-Myc over-expression is associated with more advanced and aggressive tumor phenotypes, suggesting that c-Myc plays a crucial role in the pathogenesis of HCC [1]. Solanum Lyratum, a Chinese anti-cancer herbal drug that contains tanshinone IIA, up-regulates the expression of Fas, p53, and Bax but down-regulates Bcl-2 and c-Myc in human hepatocarcinoma cell line (SMMC-7721) [35]. Tachyplesin, isolated from acidic extracts of hemocytes from the Chinese horseshoe crab (*Tachypleus tridentatus*), effectively inhibits proliferation and induces differentiation of hepatocarcinoma cells. It also up-regulates the expression of p21 and down-regulates the expression of c-myc [36].

To date, a variety of in vivo animal models has been applied to explore the potential anticancer agents. The most common tumor model used today is the subcutaneous injection of tumor cells into the paravertebral/flank region of immunocompromised mice. This method is simple to perform, is associated with a low intra-procedure mortality rate, and allows for easy visualization and monitoring of tumor growth. However, it has a low fidelity to the actual environment in which tumor cells proliferate. A capsule often forms around a tumor, with metastases rarely occurring [20]. Biochemical milieu and blood supply notably differ in the skin versus organs, especially with hepatoma, where portal system and drug-detoxifying enzymes exist [19]. In addition, both orthotopic and ectopic organ environments differentially influence the sensitivity of murine colon carcinoma cells to doxorubicin and 5-fluorouracil [37]. Orthotopic models of tumor implantation allows for the study of therapeutic effects of agents and molecular events associated with progression, metastasis, and cancer therapy [38].

The anti-cancer effects of ZJW have been reported in the subcutaneous murine xenograft models after inoculating human colon cancer HCT116 or HT29 cells into immunocompromised nude mice [16], and in the subcutaneous murine allograft models after inoculating mouse 4 T1 breast cancer cells into female BALB/c mice [18] or S180 tumor cells into Kunming mice [15]. However, the anti-cancer effects of ZJW against human HCC cells have not been studied in either nude mice or immunocompetent mice. Nowadays, different types of mouse models have been developed for liver cancer research [19]. Human or murine HCC cultured cells or tissue fragments are inoculated into immunocompromised mice (xenograft models) or immunocompetent syngeneic mice (allograft models). Several evidences have emphasized the importance of tissue microenvironment on the biological behavior of HCC xenograft or allograft, and the response to anti-cancer agents. As noted, it is strongly recommended that the potential anticancer agents assessed in ectopic implantation models of HCC need to be confirmed in orthotopic implantation models [19]. In this study, we successfully developed an orthotopic intrahepatic xenograft immunocompetent mouse model and applied this model to study the anti-cancer effects of ZJW. Immunostaining with anti-luciferase antibody confirmed that HepG2/NF-κB/Luc cells had been transplanted successfully in livers and a markedly lower number of luciferase-positive cells in ZJW-treated groups relative to control was observed, indicating that ZJW was a potent anti-cancer agent in vivo.

The anti-cancer activity of ZJW against HepG2/NF-κB/Luc cells in ICR mice was demonstrated at the dosage of 200 mg/kg of ZJW concentrated powder, equivalent to 125 mg/kg of CC and 18.9 mg/kg of ER in ZJW. This dose was lower than the dose reported in a previous study, in which ZJW inhibits tumor growth in S180-bearing Chinese Kunming mice at the dosage of 420 mg/kg [15]. However, inter-study comparisons are somewhat complicated by differences in the species of tumor origin, ectopic or orthotopic tumors, mouse strains, immune competence of recipient mice, dosing regimen, and so on. By considering the body surface area of human and mice, we further converted the ZJW dose and found that the human equivalent dose of ZJW in this study was 16 mg/kg, which was lower than the adult daily dose (450 mg/kg) of ZJW recommended by Chinese doctors. Our study showed an obvious anti-hepatoma effect of ZJW in a novel intrahepatic HepG2 xenograft immunocompetent mouse model. Further studies on clinical trials of ZJW in liver cancer patients may facilitate to explore the potential therapeutic effects of ZJW for liver cancers.

## Conclusions

In conclusion, our data showed for the first time that ZJW significantly suppressed human cancer cell growth in orthotopic HepG2 xenograft-bearing immunocompetent mice. Moreover, microarray analysis revealed that CC and ZJW displayed a similar gene expression profile in HepG2 cells. Network analysis of gene regulated by ZJW further suggested that c-myc oncogene played a critical role in the cytotoxic effect of ZJW. Further studies are needed to clarify the role of c-myc and the molecular mechanisms of growth inhibition in hepatoma cells exposed to ZJW and its constituents.

## Abbreviations

AP-1: Activator protein 1
CC: *Coptis chinensis* Franch
DMEM: Dulbecco’s modified Eagle’s medium
EIF4A2: Eukaryotic translation initiation factor 4A isoform 2
ER: *Evodia rutaecarpa* Benth
GAPDH: Glyceraldehyde-3-phosphate dehydrogenase
HCC: Hepatocellular carcinoma
HPLC: High-performance liquid chromatography
MTT: 3-[4,5-dimethylthi- azol-2-yl]-2, 5-diphenyltetrazolium bromide
NF-κB: Nuclear factor-κB
PBS: Phosphate-buffered saline
qPCR: Quantitative real-time polymerase chain reaction
SAS: Statistical Analysis Software
TC_50_: Toxic concentration at 50%
TCM: Traditional Chinese medicine
ZJW: Zuo-Jin-Wan
